# Neurovascular Reactivity in the Aging Mouse Brain Assessed by Laser Speckle Contrast Imaging and 2-Photon Microscopy: Quantification by an Investigator-Independent Analysis Tool

**DOI:** 10.3389/fneur.2021.745770

**Published:** 2021-11-11

**Authors:** Fatma Burcu Seker, Ziyu Fan, Benno Gesierich, Malo Gaubert, Rebecca Isabella Sienel, Nikolaus Plesnila

**Affiliations:** ^1^Institute for Stroke and Dementia Research, Munich University Hospital and University of Munich, Munich, Germany; ^2^Munich Cluster for Systems Neurology (SyNergy), Munich, Germany

**Keywords:** neurovascular coupling, hypercapnia, laser speckle contrast imaging, two-photon microscopy, aging, investigator-independent analysis

## Abstract

The brain has a high energy demand but little to no energy stores. Therefore, proper brain function relies on the delivery of glucose and oxygen by the cerebral vasculature. The regulation of cerebral blood flow (CBF) occurs at the level of the cerebral capillaries and is driven by a fast and efficient crosstalk between neurons and vessels, a process termed neurovascular coupling (NVC). Experimentally NVC is mainly triggered by sensory stimulation and assessed by measuring either CBF by laser Doppler fluxmetry, laser speckle contrast imaging (LSCI), intrinsic optical imaging, BOLD fMRI, near infrared spectroscopy (NIRS) or functional ultrasound imaging (fUS). Since these techniques have relatively low spatial resolution, diameters of cerebral vessels are mainly assessed by 2-photon microscopy (2-PM). Results of studies on NVC rely on stable animal physiology, high-quality data acquisition, and unbiased data analysis, criteria, which are not easy to achieve. In the current study, we assessed NVC using two different imaging modalities, i.e., LSCI and 2-PM, and analyzed our data using an investigator-independent Matlab-based analysis tool, after manually defining the area of analysis in LSCI and vessels to measure in 2-PM. By investigating NVC in 6–8 weeks, 1-, and 2-year-old mice, we found that NVC was maximal in 1-year old mice and was significantly reduced in aged mice. These findings suggest that NVC is differently affected during the aging process. Most interestingly, specifically pial arterioles, seem to be distinctly affected by the aging. The main finding of our study is that the automated analysis tool works very efficiently in terms of time and accuracy. In fact, the tool reduces the analysis time of one animal from approximately 23 h to about 2 s while basically making no mistakes. In summary, we developed an experimental workflow, which allows us to reliably measure NVC with high spatial and temporal resolution in young and aged mice and to analyze these data in an investigator-independent manner.

## Introduction

Since the brain stores very little energy, proper neuronal function depends on a constant supply of glucose and oxygen via cerebral blood flow. During increased neuronal activity, the need for nutrients increases and the necessary excess energy is delivered via a tightly regulated redistribution of blood flow to these active areas by dilation of cerebral blood vessels. This regulation of cerebral blood flow (CBF) by the crosstalk between neurons and cerebral vessels is called “neurovascular coupling” or NVC (1, 2). NVC responses can be utilized as an indicator of neuronal activity. Under pathological conditions, NVC may be used to detect dysfunctions of the cerebrovascular system (3–5).

To investigate NVC *in vivo*, three different steps are needed: (1) a stimulation paradigm, (2) a fast technique to measure CBF or to visualize cerebral vessel reactivity, and (3) an unbiased and reliable method for data analysis. The first step depends on which region of the brain is studied. Typically, odor and visual cues, electrical or tactile sensory stimulations of the whiskers or the paws are used (6–9). The second prerequisite can be achieved using non-invasive optical methods based on the speckle pattern of moving red blood cells (Laser Speckle Contrast Imaging, LSCI). LSCI is a very powerful imaging tool for the 2-D visualization of perfusion dynamics in tissues (10–12). Another imaging method for visualizing the dynamics of the cerebral vasculature is two-photon microscopy (2-PM) (13–15). 2-PM is a state-of-the-art confocal scanning microscopy technique with a high spatial and temporal resolution able to visualize pial vessels, penetrating arterioles, and deep cortical microvessels *in vivo*. The final, but equally critical step is data analysis. The software provided with commercially available LSCI units has mainly been developed for data acquisition in single human subjects and has therefore only limited analysis capabilities. This is particularly true when it comes to the analysis of data sets acquired at different time points and in whole groups of subjects. Therefore, commercially available LSCI analysis solutions are not entirely suitable for research purposes. Also analyzing images obtained by 2-PM is a time-consuming manual process prone to investigator bias. Therefore, the main aim of the current study is to present a novel tool for the analysis of LSCI and 2-PM data to establish a flexible, software-based analysis pipeline for an automated and investigator-independent analysis of the obtained data sets.

To test our new experimental workflow, we used a model of physiological aging, a paradigm well-known to be associated with an age-related decrease in NVC. The world population is aging and over 30% of the people in western countries will be older than 65 years of age by 2050 (16). Aging causes significant structural changes in brain volume, in the dendritic arbor, spine and synapse numbers, and vasculature (17, 18). Among this population, cognitive impairments related to vascular changes are responsible for at least 20% of all dementia cases (19). Surely, NVC as a key homeostatic regulator is inevitably affected by these vascular changes (20). Sensory-evoked NVC responses are indirect measures of neuronal activation and any alteration in NVC can predict underlying pathology (21, 22). A substantial number of publications from various laboratories suggests impaired NVC in aged humans and experimental animals (23–27). It has been shown that age strongly alters CBF regulation in humans, specifically steady-state CBF decreases progressively during the aging process concomitant with an increase in CBF pulsatility after midlife (28). Also, structurally, cerebrovascular pathologies involving small arteries and arterioles are very common in the aged brain (29–31). Therefore a deep understanding of how NVC changes during aging is an important prerequisite in order to decipher the mechanisms underlying cerebrovascular disease, dementia, and neurodegeneration (32–34). Aging affects the normal structure and function of the neurovascular unit (NVU). Aged mice show decreased astrocyte end-feet density, reduced pericyte coverage in the hippocampus, more activated microglia, and reduced CBF as compared to young mice (35). Moreover, aging and concomitant metabolic disorders such as obesity impair NVC in animals and humans thereby making the aging brain more vulnerable to age-related neurodegenerative disorders such as Alzheimer's and Parkinson's Disease (4, 36, 37). Therefore, deciphering how NVC is affected by aging may lead to new therapeutic strategies for these disorders.

## Materials and Methods

### Experimental Animals

All experimental procedures were conducted according to institutional guidelines of the University of Munich and were approved by the Government of Upper Bavaria (animal protocol number: Vet_2-15-196). In all parts of the experiments, 6–8 weeks old C57BL/6N mice were purchased from Charles River Laboratories (Sulzfeld, Germany). Mice at the age of 6–8 weeks were categorized as young and mice at the age of 1 year and 2 years were categorized as aged. The mice were aged in the animal facility of the Institute of Stroke and Dementia Research. All mice were housed in groups of five in isolated ventilated HEPA filtered cages with a 12-h light/dark cycle with *ad libitum* access to food and water. All cages had standard enrichment.

### Chronic Cranial Window Implantation

A chronic cranial window was implanted over the left somatosensory cortex between the coronal and the sagittal suture. The rostromedial corner of the window was placed as close as possible to Bregma. Mice received buprenorphine (0.1 mg/kg) 30 min before surgery for analgesia and anesthesia was induced with 5% isoflurane and maintained with 2% isoflurane in 70% room air and 30% O_2_ during surgery. A feedback-controlled heating pad was used to maintain body temperature at 37°C. Animals were fixed in a stereotactic frame using a nose clamp and the scalp was incised along the midline. Lidocaine (2%) was applied topically onto the skull as a local anesthetic and a round craniotomy with a diameter of 4 mm was performed above the somatosensory cortex and covered with a glass window. A plastic ring (diameter: 1 cm; weight: 0.1 g) was glued on top of the cranial window to form a water reservoir. After surgery, mice were placed in a pre-heated wake-up box (32°C) until all vital functions recovered. All mice received buprenorphine and enrofloxacin (10 mg/kg s.c.) once daily for three days after surgery.

### Whisker Stimulation

**Figure 1:**
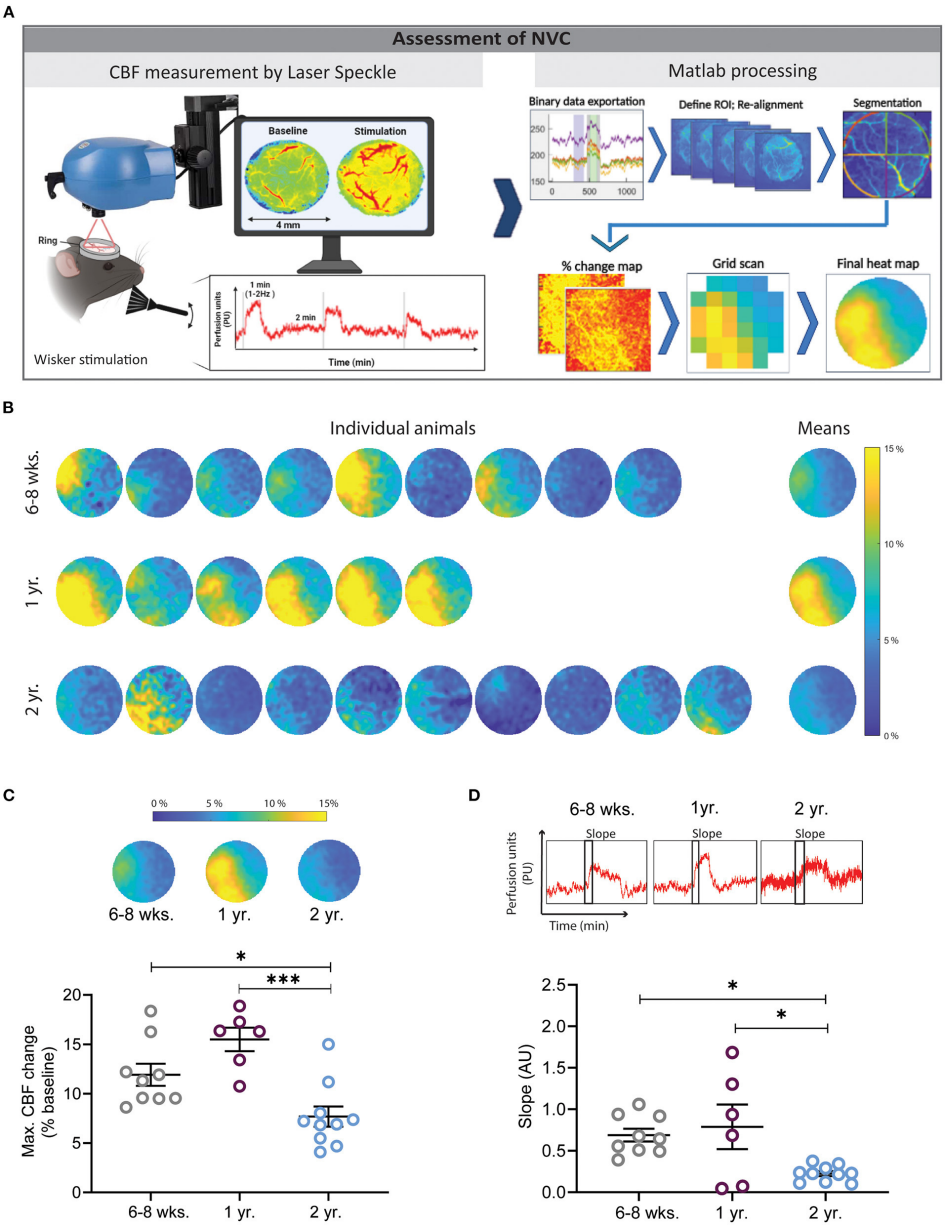
Assessment of neurovascular coupling (NVC) using laser speckle contrast imaging (LSCI) and a Matlab-based analysis pipeline. **(A)** Experimental setup for whisker stimulation and data analysis. Data visualization with LSCI shows a flow map and a perfusion trace (left), data processing steps show binary data exportation, ROI alignment, segmentation, % change map, grid preparation for quantification, and final averaged heat map (right). **(B)** Heat maps of cerebral perfusion (CBF) in individual mice of different ages following whisker stimulation. Each perfusion map was created by averaging LSCI values from three whisker stimulations. Mean depicts the average of all animals in one group. The dark blue color indicates no or small changes in cortical perfusion, while the yellow color indicates increases of cortical perfusion of up to 15%. **(C)** Quantification of maximal CBF changes. Young and 1-year-old mice had significantly higher NVC responses in comparison to 2 years old mice (*P < 0.05: 6–8 weeks vs. 2 years old, ***P < 0.001: 1 year vs. 2 year, One-way ANOVA). **(D)** Quantification of the velocity of CBF increase after whisker stimulation by slope analysis. Two year old mice had a significantly slower CBF increase in comparison to young and 1 year old mice (*P < 0.05: 6–8 weeks vs. 2 year and 1 year vs. 2 year) (*n* = 6–10 mice/group, mean ± SEM, One-way ANOVA).

Three weeks after window implantation, mice received medetomidine (0.05 mg/kg, sc) for light sedation. After 10 min animals were anesthetized with 2% isoflurane and placed in a stereotactic frame. Afterwards, isoflurane was gradually reduced to 0.5–0.75% (in 70/30% Air/O_2_) and whisker stimulation was performed over one minute by manually or mechanically stroking the contralateral (right) vibrissae with a brush at a frequency of 1–2 Hz. For 2-PM we developed a custom made motorized brush holder and use the same stimulation protocol as for the manual stimulation. The procedure was repeated three times with two min intervals (Figure 1A).

### CO_2_ Challenge

**Figure 2:**
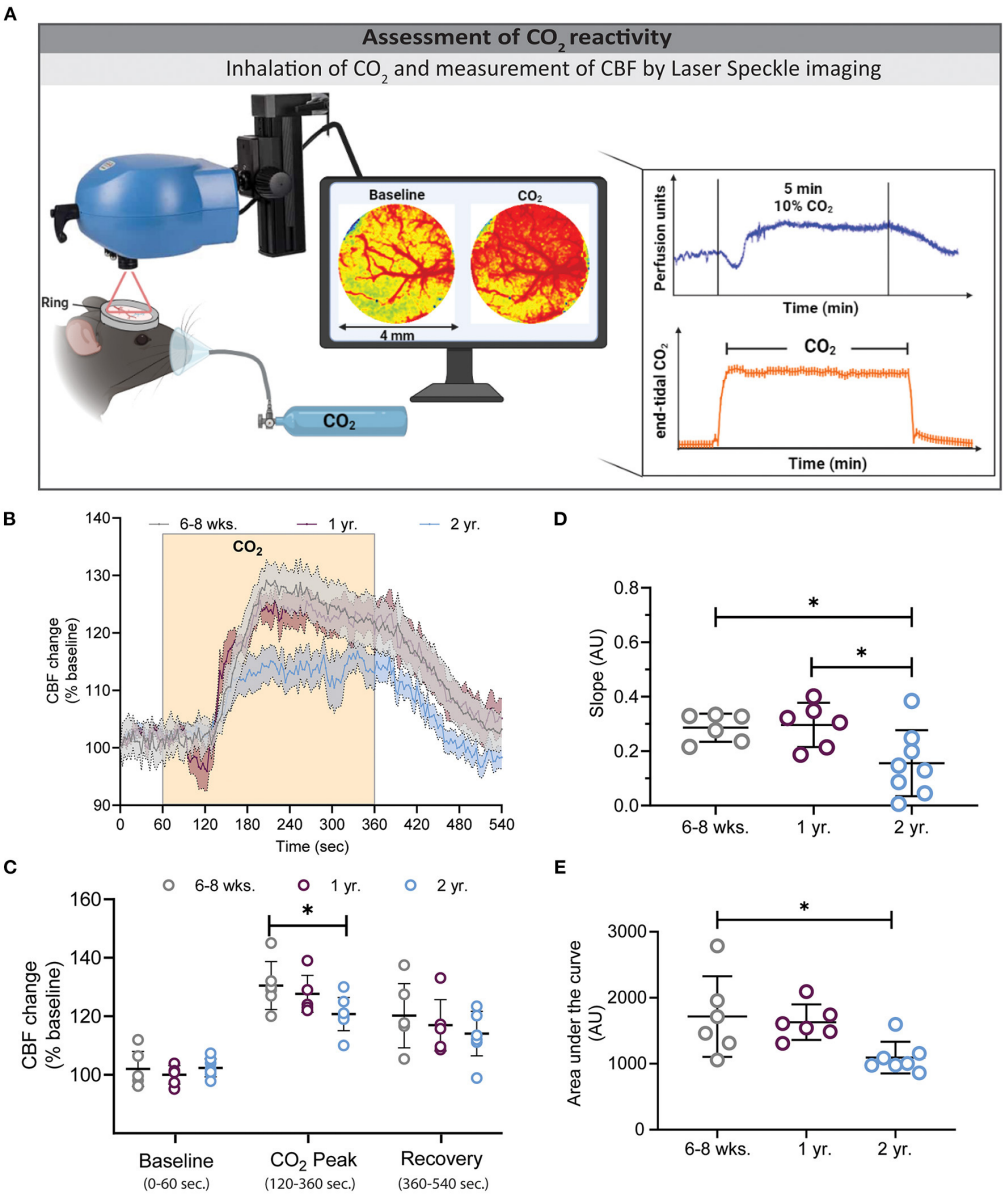
Assessment of CO_2_ reactivity using laser speckle contrast imaging (LSCI). **(A)** Experimental setup of the CO_2_ challenge (left) and exemplary traces for LSCI perfusion (top right) and end-tidal CO_2_ (bottom right). **(B)** CBF changes before, during, and after hypercapnia in the three investigated age groups. Two-year-old mice showed a low **(C)** and slow **(D)** CBF increase during hypercapnia, whereas young and 1-year-old mice had higher peak values (*P < 0.05: 6–8 weeks vs. 2 year), and faster slope changes (*P < 0.05: 6–8 weeks vs. 2 year and 1 year vs. 2 year). **(E)** Quantification of the area under the curve (AUC) of graph B depicts smaller AUC in 2 year old mice in comparison to 6–8 weeks and 1 year old mice (*P < 0.05) (mean ± SEM, *n* = 6–8 mice/group, One-way ANOVA).

To evoke hypercapnic hyperemia, mice were ventilated with 10% CO_2_ and 30% O_2_ in room air for five min (Figure 2A). End-tidal CO_2_ was measured in % with a capnograph (Figure 2A, right) and recorded using a digital data acquisition system (PowerLab, AD Instruments, Australia).

### Measurement and Analysis of Local Cerebral Blood Flow by Laser Speckle Imaging

A laser speckle contrast imager (LSCI, Perimed, Järfälla, Sweden) was positioned 10.4 cm above the chronic cranial window and a 0.5 x 0.5 mm field of the cortex was imaged at 4.4 Hz. The data was recorded using the software supplied with the device (Pimsoft, Perimed, Järfälla, Sweden) and analyzed using a custom Matlab script (MATLAB, R2016b, The MathWorks, Natick, MA). First, a spherical region around the rim of the cranial window containing the exposed cortex (ROI), was drawn manually. For each pixel within the ROI, the perfusion signal was filtered with a cut-off frequency of 0.004 Hz to remove any signal drift and allows re-alignment of the acquired frames. This process is important while the brain and cerebral vessels move slightly due to heart beat and breathing. Hence, when using high resolution imaging, e.g., LSCI or 2-PM, images acquired at different time points shift slightly. Therefore, re-alignment is necessary to correctly allocate measurements obtained over time to exactly the same pixel. This filter was implemented using the MATLAB functions cheby1 and filtfilt to design a Chebyshev Type I filter of order 2 and to perform zero-phase digital filtering. Then a threshold was defined using Otsu's method, to detect stimulation periods automatically. The correct detection of stimulation periods was verified visually. To account for a potentially ramp-like increase of the perfusion signal at the beginning of the stimulation, the perfusion signal was averaged within 10 and 30 s after the automatically detected stimulation onset and normalized to the baseline perfusion signal, defined individually for each stimulation period as the average signal within 40 to 10 s before the automatically detected stimulation onset. The hereby resulting, normalized response of the perfusion signal to the stimulation was then averaged across stimulation periods. First individually for each animal and then across animals within each experimental group. To allow averaging across animals, the images, cropped around the spherical ROI, were resized to an image matrix of 120 x 120 pixels. For a better understanding of the individual responses, heat maps were also acquired for individual animals (Figure 1B).

### Measurement and Analysis of Vessel Diameter by *in vivo* Two-Photon Microscopy

**Figure 3:**
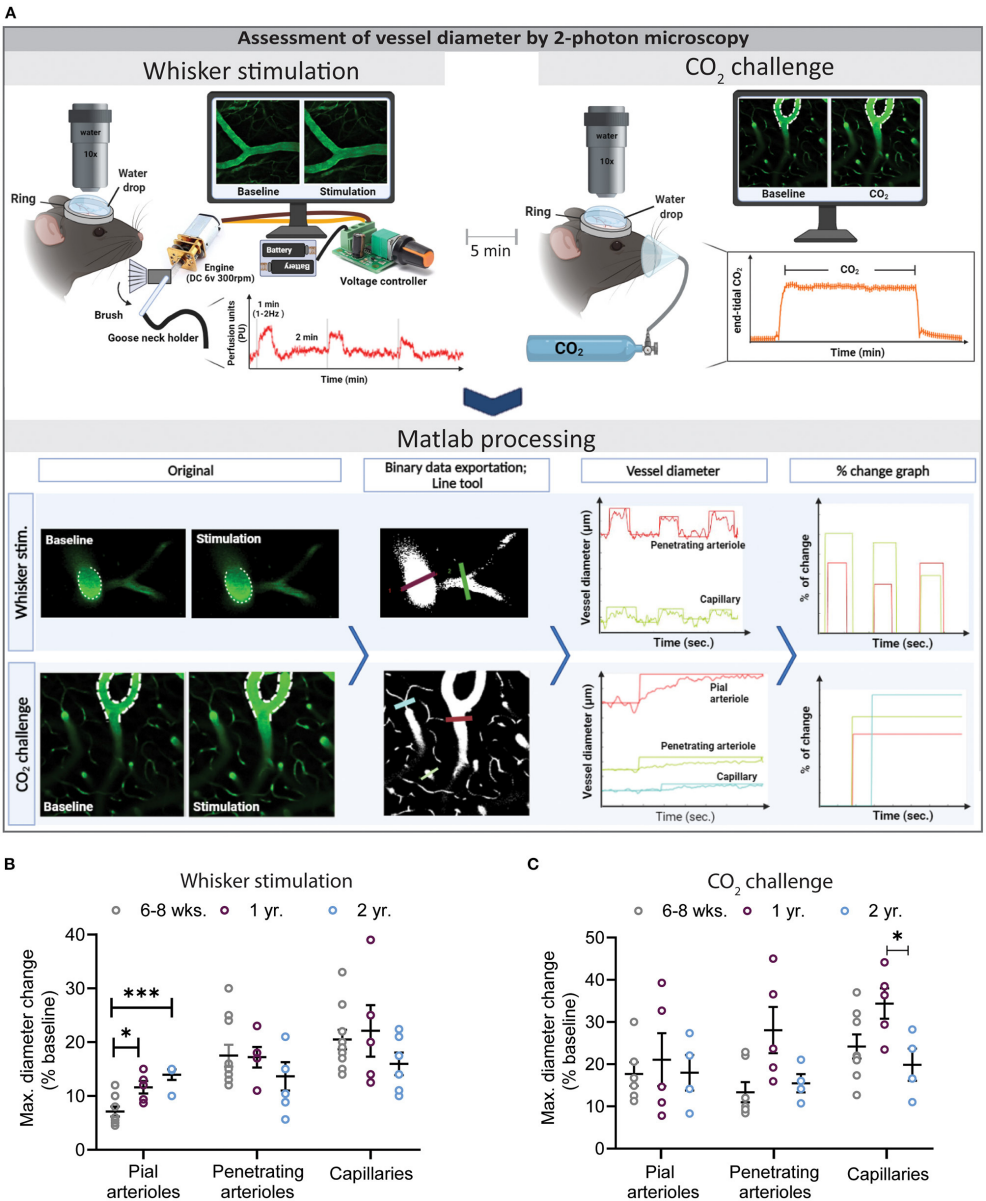
Assessment of vessel diameter by 2-photon microscopy. **(A)** Experimental setup for whisker stimulation (top left) and hypercapnia during (top right) and a schematic drawing of the data analysis work flow by the newly developed Matlab script. **(B)** Quantification of maximal diameter changes in different vessel segments at different ages following whisker stimulation. Pial vessel reactivity increased with age (*P < 0.05: 6–8 weeks vs. 1 year and ***P < 0.001: 6–8 week vs. 2 year) while over all the capillary response was reduced in 2-year-old mice in comparison to young and 1-year-old mice. **(C)** Quantification of maximal diameter changes in different vessel segments at different ages following hypercapnia. Neurovascular reactivity of cerebral capillaries was impaired in 2-year-old mice as compared to 6–8 weeks and 1-year-old mice (*P < 0.05: 1 year vs. 2 year) (mean ± SEM, *n* = 4–10 mice/group, One-way ANOVA).

Two-photon microscopy (2-PM) was performed the day after the LSCI imaging using the same anesthesia protocol as described above. For visualizing the cerebral vasculature 0.1 ml of fluorescein isothiocyanate (2,000 kDa) was injected through the tail vein using a mouse tail illuminator (Braintree Scientific, USA). Then mice were transferred under the 2-PM. Pial and parenchymal vessels in the region of the barrel cortex were visualized as time series videos (2 s per frame) at a depth of 50–100 μm with a 10x Zeiss EC Plan—NeoFluar objective using a Li: Ti laser tuned to 800 nm. The whisker stimulation protocol was followed by 5 min 10% CO_2_ challenge. A custom-made automated brush holder was used for whisker stimulation while imaging with 2-PM (Figure 3A). For this device, a small brush and a voltage controller for frequency adjustments were mounted to a 300 rpm (6 V) battery-powered motor (Walfront, China). A gooseneck holder was used to fix the motor on the imaging platform, which allowed fine-tuning the angle of the brush.

Vessel diameter analysis was performed using a custom-made Matlab routine (MathWorks version R2020a, Natick, Massachusetts, USA; www.mathworks.com) based on imshow3D developed by Maysam Shahedi (https://www.mathworks.com/matlabcentral/fileexchange/41334-imshow3d) and on a Zeiss LSM file reader developed by Cy Y (Zeiss Laser Scanning Confocal Microscope LSM file reader; https://github.com/joe-of-all-trades/lsmread). First, all images have been inspected and the ones containing artifacts (heavy motion between frames, low signal-to-noise ratio) were removed. Then, time series videos were binarized, and diameter and percent change graphs were displayed. Then, line segments were drawn by the user between the vessel walls. Every time a line was drawn, the diameter of the vessel in μm along time frames were calculated by counting the number of voxels with a value of 1 (“1” corresponds to a vessel, while “0” to the background) intersecting with the drawn line and multiplied by the voxel size. Of note, some erythrocytes passing through the vessel are reducing the intensities of specific voxels, which could lead to underestimation of vessel diameter. To minimize this effect, the thickness of the line was defined to 4 voxels and the computation of vessel diameter was thus adapted by averaging the lines around the drawn line. Based on the computation of vessel diameters along time, output graphs are displayed and updated for each new line and depicted with the same color as the line drawn on the image (Figure 3A, lower left). Moreover, automatic detection of percentage peak change was provided thanks to the Matlab routine. This peak detection was based on three parameters defined in the graphical interface. Briefly, the algorithm first computed the baseline diameter by averaging a predefined number of initial frames (no stimulation). For the peak detection, the difference between the subsequent frames (with stimulation), and the baseline was calculated. After the end of a peak, a new baseline was computed, and the peak detection continued in the subsequent frames. The detected peaks were then displayed in the first graph, in the same color as the line drawn on the image (see screenshots, Figure 3A, lower right). The slope of dilation between the maximal vessel diameter of a detected peak and related baseline was also calculated automatically using the following formula:


$$\begin{array}{l} \text{Slope}=\frac{diameter_{max}-diameter_{baseline}}{time_{max}-time_{baseline}} \end{array}$$

with *diameter*_max_, *time*_max_, the diameter of the maximal value in the peak and the peak time, and *diameter*_*baseline*_, *time*_*baseline*_, baseline diameter and the time on when this baseline was calculated (last timepoint before the detection of the peak). This slope was then normalized by the baseline vessel diameter. Finally, the percent of change was calculated by the Matlab routine as the difference between the maximal vessel diameter of a detected peak and related baseline as:


$$\begin{array}{l} \text{Percent change}=\frac{{diameter}_{max}-\;diameter_{baseline}}{diameter_{baseline}}\times \;100 \end{array}$$

and displayed. For each drawn segment, all vessel diameters along time and information on peaks (start, end, slope, percentage of change) have also been exported in a table for subsequent statistical analyses.

### Data Availability

Scripts will be shared upon request.

### Statistics

Statistical analyses were performed with GraphPad Prism 7.0 software (GraphPad Software, San Diego, California USA). First, data were tested for normal distribution. Student *t*-test was used to compare two sets of normally distributed data. Multiple groups were compared with one-way ANOVA or one-way ANOVA on ranks depending on the presence or absence of normal distribution or two-way ANOVA for repetitive measurements. Differences with *P* < 0.05 were considered statistically significant.

## Results

In this study, we developed a fast, reliable, and unbiased data processing tool for the analysis of neurovascular reactivity (NVC and CO_2_ inhalation) assessed by LSCI and 2-PM. While LSCI provides information about CBF changes in the superficial layers of the cerebral cortex, *in vivo* 2-PM imaging directly visualizes diameter changes of individual cerebral vessels. For the validation of our newly developed analysis tools, we used aging as a model.

### Decreased Neurovascular Coupling in Aged Mice

Mice were given three subsequent whisker stimulations (Figure 1A). All LSCI data were first acquired with Pimsoft software^®^. After the acquisition, data were extracted in binary format (.dat) and processed with a custom-made Matlab tool. The results were exported to a spreadsheet file and further analyzed and plotted. Furthermore, the Matlab tool generated an averaged image of all three stimulations, created CBF heat maps of individual mice, and allowed to average CBF values of all investigated mice in one single heat map (Figure 1B). The heat maps show an increased CBF in the area of the somatosensory cortex whereas CBF remained unchanged in the unstimulated surrounding cortex. CBF values from the heat maps could be extracted as numerical data and plotted. Two year-old mice had a 46 and 59% lower CBF response as compared to young and 1-year-old mice, respectively (Figure 1C, *P < 0.05: young vs. 2 year old, ***P < 0.001: 1 year vs. 2 year). No significant difference was found between young and 1-year-old mice. Moreover, 1-year-old mice had a moderately higher NVC response in comparison to young mice (Figure 1C). The slope of the CBF peak after the NVC stimulation was analyzed and a reduced slope was found in 2-years-old mice in comparison to young and 1-year-old mice, respectively (Figure 1D, *P < 0.05: young vs. 2 year and 1 year vs. 2 years), indicating a slower response in this age group. No difference was found between young and 1-year-old mice (Figure 2D).

### Decreased and Sluggish CO_2_ Reactivity in Aged Mice

Following whisker stimulation, animals received 10% CO_2_ by inhalation to induce hypercapnia and subsequent cerebral hyperemia. End tidal pCO_2_ was assessed by microcapnometry and CBF was measured by LSCI (Figure 2A). A typical CBF response consists of an increase in CBF within one min after CO_2_ inhalation followed by a plateau phase and a gradual recovery phase after termination of CO_2_ inhalation (Figure 2B). Two-year-old mice showed a slower and lower CO_2_ response curve in comparison to young and 1-year-old mice [*F*_(280, 2380)_ = 2.329, ***P < 0.0001, 2-way ANOVA]. The peak response in 2-year-old mice was reduced by 31 and 25% in comparison to young and 1-year-old mice, respectively (Figure 2C, *P < 0.05: young vs. 2 year). Not only the degree, but also the velocity of the CBF response was reduced in 2-year-old mice as indicated by a reduced slope of the CBF increase (Figure 2D, *P < 0.05: young vs. 2 year and 1 year vs. 2 year). As an integrative measure of the whole CBF response after hypercapnia we calculated the area under the curve (AUC). As expected from the previous measurements, also this value was significantly reduced in 2-year-old mice (Figure 2E, *P < 0.05: young vs. 2 year and 1 year vs. 2 year).

### Reduced Vasodilation During NVC and Hypercapnia in Aged Mice

To directly visualize vascular reactivity in young, adult, and aged mice, animals received the plasma label FITC dextran and cerebral microvessels we imaged by *in vivo* 2-PM. Time series were recorded with the Zen^®^ software (Zeiss, Oberkochen, Germany). Afterwards, original files were processed with our novel, investigator-independent vessel diameter measurement script and diameter changes of different vessel segments (pial arterioles, penetrating arterioles, and capillaries) were analyzed. A straight line crossing the investigated vessel at an angle of 90° was drawn and the inner diameter of the vessel was assessed based on the fluorescence of the injected plasma marker. Absolute changes of vessel diameter following whisker stimulation or hypercapnia were calculated by the script and expressed as % baseline (Figure 3A). A total of three whisker stimulations and one CO_2_ challenge were performed and analyzed.

The most pronounced vasodilation was observed in capillaries independent of the age of the animals or the stimulation paradigm (Figures 3B,C). Two-year-old mice showed a reduced response in penetrating arterioles and in capillaries, specifically after hypercapnia (Figure 3C, *P < 0.05: 1 year vs. 2 year), suggesting a spatially distinct effect of age on cerebrovascular reactivity.

## Discussion

In the current study, we developed two Matlab-based software tools, the “NVC-ToolBox,” to analyze neurovascular reactivity by *in vivo* LSCI and 2-PM imaging following whisker stimulation and hypercapnia. The NVC-ToolBox allowed us to analyze neurovascular reactivity in a fully blinded, automatized, fast, and user-friendly manner. Moreover, we used aging as a paradigm to validate the NVC-ToolBox, since aging is well-known to reduce neurovascular reactivity. Our results demonstrate that the NVC-ToolBox is an easy to use and reliable tool for the assessment of neurovascular reactivity. Statistically significant changes in neurovascular reactivity between groups could be identified with a group size of six animals and a reasonable variability. Further, the time needed to analyze the data could be shortened by over 90% and investigator bias was completely eliminated from the analysis process. In the manual analysis procedure, first, frames of 2-PM time series must be exported as separate images which is between 250 and 280 frames. Then these images are binarized, skeletonized and diameters were measured by another plugin using ImageJ software. This procedure takes for the manual analyzer approximately 5 min for each image. However, with the automated tool it takes only 1–2 s to analyze all frames. Therefore the differences between the manual and automated analysis in sense of time and quality is massive. In terms of the effect of age on neurovascular reactivity, our data suggest a differential response during aging. Further, 2-PM imaging revealed a distinct involvement of different vessel segments following NVC and hypercapnia, i.e., the most pronounced vasodilation was found primarily at the capillary level and aging reduced vascular reactivity mainly in this vascular bed; to our surprise pial arteriole reactivity following whisker stimulation was even increased during the aging.

NVC ensures rapid CBF regulation in response to neuronal activity. *In vivo* imaging techniques with a high spatiotemporal resolution are crucial for understanding changes in NVC, a process critical in many pathologies such as aging or dementia. Moreover, reliable assessment of NVC is only possible with proper surgical window preparations, stable animal physiology, reliable and reproducible stimulation modalities, precise data acquisition, and objective image analysis algorithms (38). One of the gold standard techniques for NVC measurements is LSCI which is a simple, non-invasive *in vivo* imaging technique for the measurement of tissue perfusion (39–43). When moving objects are illuminated by dispersed laser light, the scattered light will form an interference (“speckle”) pattern, which is proportional to tissue perfusion (44, 45). This technique is well-established and frequently used for dynamic imaging of CBF thanks to its high temporal resolution (11). When NVC is induced by forepaw, hind paw, or whisker stimulation, cerebral arterioles in the respective cortical area dilate to redistribute blood flow to the activated area. The subsequent increase in CBF can be visualized by 2-D heat maps using LSCI. Commercially available LSCI devices display, store and allow analyzing heat maps of individual animals, however, lack modalities to analyze data from cohorts of animals, specifically longitudinally. Since CBF responses can be spatially distinct and vary in intensity, changes often became only apparent when individual values of a group of animals are averaged or superimposed. This approach termed “co-registration” is not completely novel, since it has already been successfully used for the analysis of large cohorts of human and animal MRI, BOLD, or PET data (46, 47), however, it has, to the best of our knowledge, never been employed for the analysis of LSCI data sets. Using the NVC-ToolBox, multiple stimulations from the same subject and multiple subjects from the same experimental group can be averaged and high-resolution images displaying the mean response are created in a fully blinded and automated manner. Using this novel analysis tool, we observed that following whisker stimulation, young mice show a focal CBF response in the middle of the whisker area, while, 1-year-old mice showed a higher response covering almost the whole somatosensory cortex. In 2-year-old mice the response was focused again, but weak (Figure 1B). The observed changes in the spatial distribution of the laser speckle recordings could be linked to different factors. We can speculate that either the vascularization or the vascular function (or both) change with age. Many laboratories reported a reduction of capillary number and density in the aged brain (48–51). Others reported a substantial age-related decrease in brain arteriolar density in 24 vs. 13 months old rats (52–54). Some studies suggest that age-related changes can be multiphasic in the sense that capillary density increases during late adulthood and then declines at advanced ages in humans and rats (55, 56). Li et al. suggested a reduced capillary density together with a higher capillary flow velocity and heterogeneous capillary flow pattern in older vs. younger mice by using optical coherence tomography angiography (57). Therefore, oxygen delivery may be reduced during aging (58). Despite these data on vessel density and vessel function, quite little is known about cerebrovascular plasticity during the aging process. Our data suggest that aging is also associated with significant changes in the spatial CBF response, i.e., that aging induces plastic changes of the neuronal network, which is then followed, by a consecutive vascular change. Further experiments using longitudinal imaging in individual animals may answer this important issue.

Two-photon laser scanning microscopy is a state-of-the-art technique used for observing, and measuring vascular changes *in vivo* with high spatial and temporal resolution with deep tissue penetration (59). In the current study, we used 2-PM to visualize changes of the vascular diameter of different vessel segments upon whisker stimulation or hypercapnia. After acquiring 3-D image stacks from the cerebral cortex, the diameter of vessels needed to be measured in a reliable and efficient manner, since manual analysis of 2-PM data is a tedious, time-consuming, and quite subjective process. To overcome these shortcomings, we developed scripts to measure vessel diameter changes of all vessels present in the 3D stack quickly and reliably. The scripts used for the analysis of LSCI data, as well as the analysis of 2-PM data resulted in statistically significant changes in neurovascular reactivity between groups with a group size of six animals and reasonable variability. In addition, the time needed to analyze the data could be shortened significantly and investigator bias was abolished from the analysis process.

Another advantage of the currently used experimental approach is the use of two different imaging modalities investigating two different aspects of neurovascular reactivity. Two-photon microscopy allows the assessment of vessel reactivity with high spatial and temporal resolution, however, only in a very limited area of the cortex (e.g., 200 x 200 μm), while LSCI has a limited spatial resolution and depth penetration, but is able to assess CBF responses in much larger cortical areas (e.g., 4 x 4 mm or more). Hence, using these two techniques consecutively on the same animal has complementary advantages and will allow a more in depth understanding of neurovascular reactivity in the healthy and diseased brain.

Aging has multiple effects at the systemic, molecular, and cellular level and impairs, among others, cerebrovascular reactivity (8, 26, 27). In the current study, we performed whisker stimulation in three different age groups, namely young (6–8 weeks), 1-year, and 2-year-old mice. Our LSCI analysis tool successfully produced superimposed stimulation responses from individual mice and created high-resolution mean heat maps by averaging the data from a whole group of mice. After the creation of the maps, the visual data was processed with our tool to create numerical values. Thus, we observed a trend toward more pronounced neurovascular reactivity during the first year of age and significantly lower and slower responses in 2-year-old mice. These results are similar to those published by other laboratories, e.g., Park and colleagues also found a significant reduction of NVC in 2-year-old mice (27). Moeini et al. showed no significant tissue pO_2_ changes to whisker stimulation at the age of 15–16 months but a significant reduction at the age of 26–28 months (60). On the other hand Tucsek et al. (61) found a decreased NVC response at the age of 7 months by using laser doppler. Soleimanzad et al. also revealed a decreased response to odor stimulation at the age of 10 months by using multiexposure speckle imaging (36). Various results can be related to measurement techniques and response of specific cortex regions to specific stimuli can be differently affected during aging (60). Thus, the NVC-ToolBox generated data in line with the current literature.

Besides whisker response, 2-year-old mice had a reduced and slow vessel dilatation following hypercapnia, while 1-year-old mice showed no pathological response (Figure 2B). This data suggests that at the age of 1 year maximal dilation potential of the vessels maintain their ability to fully dilate while this function is significantly impaired by aging. Munting et al. showed no significant differences between young and 1-year-old mice to a 7.5% CO_2_ challenge by arterial spin labeling (62). In the current study we recorded a ~30% CBF increase following hypercapnia in young mice. Other studies, using different imaging techniques and anesthesia protocols, recorded values from 20 to 60% (8, 62, 63). These differences between laboratories emphasize the necessity for standardized and reproducible protocols for the assessment of neurovascular functions.

The analysis of vessel diameters at a depth of up to 100 μm within the cerebral cortex by 2-PM showed an increase in vascular reactivity toward smaller vessels, i.e., the highest response at the level of cortical capillaries. This observation was most pronounced in 1-year-old mature mice and almost eliminated in aged mice. Overall, neurovascular reactivity was reduced in 2-year-old mice in almost all vascular beds, except in pial vessels. This data suggest that different vessel segments react differently to sensory stimuli and that functional deterioration of the capillary bed might be the primary reason for reduced neurovascular reactivity and loss of vascular integrity during aging process (54). In fact, it has been reported that the tortuosity of the whole neurovascular tree (middle, anterior and posterior cerebral arterioles, penetrating arterioles, and capillaries) increases with age in mice and humans (64–67). Hence, such anatomical changes may well be part of the explanation why the functionality of the neurovascular network decreases with age. Moreover, distinct structural and functional characteristics of different vessel segments may explain why aging may have a differential effect on specific vascular beds. While pial arteries have a thick layer of smooth muscle cell lining and elastic lamina, penetrating arterioles have a thin smooth muscle layer, which gets completely lost while they dive deeper into the parenchyma. At the cerebral capillary level, endothelial cells are surrounded by a basement membrane, pericytes, and astrocytic end-feet (2, 68). Hence, these structures may be differentially affected by aging and may thus cause the observed spatially distinct neurovascular dysfunction.

Next to the degree and speed of neurovascular reactivity in different vascular beds, our data also shed some light on the signal transduction occurring along the vascular tree during NVC. A recent study from Rungta et al. showed that following neuronal stimulation blood velocity increases first in the surrounding capillary bed and only somewhat later in penetrating and pial arterioles (69), suggesting that capillaries trigger signals which are transferred to upstream arterioles and recruit them to the coupling response. Consequently, if the capillary response to neuronal activation is disturbed, also the upstream response should be reduced or interrupted. Interestingly, we did not observe this behavior in our current experiments. On the contrary, aging reduced the reactivity of cortical capillaries, but caused a hyperactive pial response, suggesting that aging may reduce the interaction between the brain parenchyma and cerebral vessels, but that upstream signaling along the vascular tree may remain intact. Hence, reduced capillary dilatation and subsequent lack of tissue perfusion would elicit increased upstream signaling and a hyperactive pial response. Investigating the mechanisms responsible for these changes, such as paravascular nerve fibers, perivascular astrocytes, adenosine, or neuronal nitric oxide (NO) (70–72), may help to reduce age related neurovascular dysfunction. Perivascular astrocytes, for example, which connect NO producing neurons with pial arterioles, are heavily activated in the aged brain (71, 73, 74). Thus, it may be speculated that activated astrocytes trigger dilation of pial arterioles in the presence of an impaired capillary response. On the other hand, pial arterioles are richly innervated by extrinsic perivascular sympathetic and parasympathetic postganglionic neurons (20, 75). Thus, it is possible that in the aged brain loss of cholinergic innervation or noradrenergic signaling could also reveal distinct pial arteriole responses in the aged brain (54, 76). Further experiments using the current developed setup may help to answer this and similar questions in the future.

Another question is of course which cellular or molecular mechanisms are responsible for the observed impairments of neurovascular reactivity during aging. Sufficient supply of the brain parenchyma with blood depends on an intact communication between the cells in the NVU, i.e. endothelial cells, astrocytes, and pericytes. It is well-known that during aging, elements of the NVU start to deteriorate resulting in dysfunction of the blood-brain barrier and capillary dilation (77–82). Reasons for these functional impairments maybe a decline in microvascular remodeling, i.e., the missing replacement of aged cells of the neurovascular unit (83), such as pericytes (80) due to the increased production of reactive oxidative species (ROS) in the aging brain (26, 84, 85) as suggested by Fan and colleagues who found significantly increased ROS production in 2-year-old mice and human brain tissue accompanied by reduced capillary density and cognitive decline (86).

In summary, we measured the effect of aging on neurovascular reactivity using two different imaging modalities and a custom-made, investigator-independent analysis tool. We show increased neurovascular reactivity during brain maturation and reduced neurovascular reactivity during aging. Furthermore, we showed that aging does not affect all cortical vessel segments equally, but has a distinct effect on capillaries. Thus, the NVC-ToolBox turned out to be a reliable, robust, and investigator-independent tool to analyze neurovascular reactivity in the healthy and aged mouse brain.

## Data Availability Statement

The raw data supporting the conclusions of this article will be made available by the authors, without undue reservation.

## Ethics Statement

The animal study was reviewed and approved by Government of Upper Bavaria (animal protocol number: Vet_2-15-196).

## Author Contributions

FS: manuscript preparation, data analysis, figure preparation, and supervised the findings of this work. ZF: carried out the experiments and data analysis. BG: LSCI image processing script writing. MG: 2-PM vessel analysis script writing. RS: figure preparation and proofreading. NP: designed the study, supervised the findings of this work, manuscript writing, and proofreading. All authors contributed to the article and approved the submitted version.

## Conflict of Interest

The authors declare that the research was conducted in the absence of any commercial or financial relationships that could be construed as a potential conflict of interest.

## Publisher's Note

All claims expressed in this article are solely those of the authors and do not necessarily represent those of their affiliated organizations, or those of the publisher, the editors and the reviewers. Any product that may be evaluated in this article, or claim that may be made by its manufacturer, is not guaranteed or endorsed by the publisher.
